# General practitioners who never perform Pap smear: the medical offer and the socio-economic context around their office could limit their involvement in cervical cancer screening

**DOI:** 10.1186/s12875-019-1004-x

**Published:** 2019-08-15

**Authors:** Chiara Maj, Lorraine Poncet, Henri Panjo, Arnaud Gautier, Pierre Chauvin, Gwenn Menvielle, Emmanuelle Cadot, Virginie Ringa, Laurent Rigal

**Affiliations:** 1 General Practice Department, Univ Paris-Sud, Le Kremlin Bicêtre, France; 2 CESP (Centre for Research in Epidemiology and Population Health), Inserm U1018, University of Paris-Saclay, University of Paris-Sud, UVSQ, Gender, Sexual and Reproductive Health Team, Paris, France; 3 Ined, Paris, France; 4 Sorbonne Université, INSERM, Institut Pierre Louis d’Épidémiologie et de Santé Publique, Department of Social Epidemiology, Paris, F75012 France; 5 Santé Publique France, Saint-Maurice, France; 6 IRD - Hydrosciences UMR 5569, Montpellier University, Montpellier, F-34090 France

**Keywords:** Cervical Cancer screening, General practitioner, Pap smear, Disparities in healthcare accessibility

## Abstract

**Background:**

In France, with the growing scarcity of gynecologists and a globally low and socially differentiated coverage of cervical cancer screening (CCS), general practitioners (GPs) are valuable resources to improve screening services for women. Still all GPs do not perform Pap smears. In order to promote this screening among GPs, the characteristics of physicians who never perform CCS should be more precisely specified. Besides already-known individual characteristics, the contextual aspects of the physicians’ office, such as gynecologist density in the area, could shape GPs gynecological activities.

**Methods:**

To analyze county (*département*) characteristics of GPs’ office associated with no performance of CCS, we used a representative sample of 1063 French GPs conducted in 2009 and we constructed mixed models with two levels, GP and county.

**Results:**

Almost 35% (*n* = 369) of the GPs declared never performing CCS. GPs working in counties with a poor GP-density per inhabitants were more likely to perform CCS (odds ratio (OR) = 0.52 for each increase of density by 1 GP per 10,000 inhabitants, 95% confidence interval (CI) = 0.37–0.74). On the contrary, GPs working in counties with an easier access to a gynecologist were more likely not to perform CCS (OR = 1.06 for each increase of density by 1 gynecologist per 100,000 women, 95%CI = 1.03–1.10 and OR = 2.02 if the first gynecologist is reachable in less than 15 min, 95%CI = 1.20–3.41) as well as GPs working in areas with a poverty rate above the national average (OR = 1.66, 95%CI = 1.09–2.54). These contextual characteristics explain most of the differences between counties concerning rates of not performing CCS.

**Conclusions:**

Specific programs should be developed for GPs working in contexts unfavorable to their involvement in CCS.

## Background

Despite the existence of an effective screening test which permits an early detection and improve chance of survival [1], there were 24,380 deaths attributable to cervical cancer in Europe in 2012, including 1170 deaths in France [2]. Guidelines in many European countries recommend that women had a Pap smear every three to 5 years [3]. In France there was no nationwide organized screening program for cervical cancer screening (CCS) before 2019 but few programs involving ambulatory care and organized at the county (*département*) level (see Table 1. for detail on the organization of CSS in France) [4, 5]. Gynaecologists performed more than 80% of Pap smear while general practitioners (GPs) performed around 10% of them in 2010 [6]. Less than 60% of French women were up to date for this screening in 2006–2008 [7] and screening participation remain highly socially differentiated to the disadvantage of those at the bottom of the social ladder [8–10]. GPs are in regular contact with the entire population, contrary to specialists, to whom the lower levels of the social hierarchy have limited access [11]. They are thus in a favourable position to offer Pap smear to underprivileged women never or rarely screened and who present the majority of invasive cervical cancers [12]. For these reasons, a higher involvement of GPs in CCS as is the case in other countries such as Denmark, the Netherlands or the United Kingdom, could lead to an increase in CCS coverage and a decrease in CCS inequalities. However not all GPs provide Pap smear [13]. In order to promote the screening among GPs who never perform Pap smear, the characteristics of these physicians should be more precisely specified.

**Table 1:** Organization of cervical cancer screening in France

● In France, the National Authority for Health (*Haute Autorité de Santé*) recommends one cervical cancer screening every three years following two normal screenings performed over two years, concerning all sexually active women aged 25 to 65 years. ● Before 2019, cervical cancer screening was mostly opportunistic. Opportunistic screening coexisted with organized screening trial programs carried out in up to 11 counties (*départements*) out of 96 in metropolitan France. Apart from the administrative staff responsible for sending invitations to overdue women, there was no medical structure or staff dedicated to organized screening. Whatever the type of screening (opportunistic or organized), women could be screened at their convenience by any of the following health professionals (by decreasing order of volume of activity): gynecologists, general practitioners, hospital-based gynecologists, midwives (whose activity is growing since its beginning in 2009 but is still scarce at the moment) and medical biologists (doctors working in outpatient medical analysis laboratories). If there was no exclusivity between these professionals regarding screening, territorial conflicts existed leading to a lack of coordination. ● Since 2019, organized screening has been implemented at the national level on the model of previous trial programs. Opportunistic screening continues to exist and will remain available. ● Regardless of the period considered, payment has remained the same for women. Within opportunistic screening, the medical consultation (where a Pap smear is performed or prescribed for sampling at the medical analysis laboratory) and the Pap smear itself are covered by the National Health Insurance (NHI). Patients pay out-of-pocket before being reimbursed (70 % of the amount covered by the NHI). Around 80% of Pap smears are performed by gynecologists and most of them charge more than what is covered by the NHI, leaving patients with out-of-pocket expenditures. In the organized screening program, Pap smears are free (sampling and analysis) without advance payment. However, medical consultation (necessary to access screening) is covered by the NHI as routine care (i.e. as in opportunistic screening), in effect not removing the financial barrier to access screening.	

Being a woman [14, 15] and working in a group practice [16] are examples of individual characteristics associated with GP’s provision of screening. Some other characteristics such as age [13, 17, 18] are inconsistently associated with the performance of CCS, and others such as practicing complementary and alternative medicine (CAM) have never been studied. We assumed that GPs who practice CAM (and particularly who practice it regularly) may have a different organization and might less often be the referring physicians for their patients. They might be perceived by patients more as a medical specialist than a GP. Their medical office might also be set up differently with no equipment to perform a gynecological examination and Pap smear.

Besides individual characteristics, the contextual aspects of the physicians’ office, like the density of gynaecologists, could also shape GPs’ gynaecological activities. The most frequently tested contextual characteristics, working in a rural area, is inconsistently associated with GP’s involvement in the screening [16, 19]. Even if the socio-economic level of the neighbourhood and the primary care supplies of the physician office could also modify GPs’ gynaecological activities, very few studies focusing on contextual aspects have been conducted and only two used a representative nationwide sample [20, 21]. They both have been conducted in the US where GPs were more likely not to perform Pap test when they practiced in metropolitan areas with population higher than 5 million. Authors suggest that this was related to the high gynaecologist density in these areas.

This article analyzes county characteristics of GPs’ office associated with GP performance of CCS, taking into account GPs’ individual characteristics. We assumed that GPs’ performance of CCS is influenced on the one hand by GPs’ personal and professional characteristics and on the other hand by social environment and health care availability and access in the counties where GPs’ offices are located. For example, GPs practicing in a county with fewer gynaecologists will be less likely not to perform CCS (because there is no gynaecologist to do it). Conversely, GPs in a county with fewer GPs and in a county with lower socioeconomic population will be more likely not to perform CCS (because they have other medical problems to manage first).

## Methods

### Data

We used data from the 2009 GP Health Barometer [22]. GP Health Barometer is a nationally representative telephone survey conducted every 3 years by the French National Institute for Health Prevention and Education (INPES), targeting GPs in private practices in France. These surveys collect GPs’ characteristics and inquire about self-reported prevention opinions and practices. The participation rate of the 2009 GP Health Barometer was 57.1%, that is 2083 GPs. In order to limit the time spent by each GP to answer the questionnaire while maintaining a large number of themes, some modules as the one about cancer screening were randomly asked to only one half of this total sample, that is 1063 GPs. GPs characteristics of both samples were compared to national average without showing any significant differences [22, 23].

### Variable of interest

**Fig. 1:**
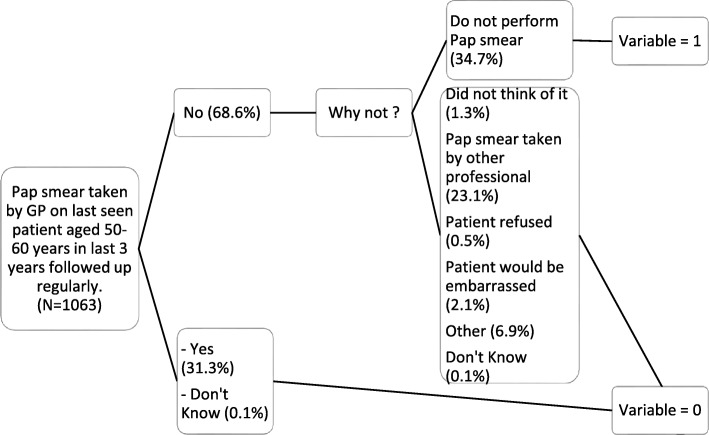
Construction of the variable ‘never performing Pap smear’ (*N* = 1063, used as denominator in percentages)

We focused on GPs declaring that they never take Pap smear. We constructed the variable ‘never performing Pap smear’ based on two existing variables. In the interviews, GPs were asked if they had themselves performed a Pap smear in the past 3 years on the last female patient seen in their practice aged 50 to 60 years. When they answered ‘yes’ or ‘I don’t know’, we coded 0. When they answered ‘no’, they were asked for what reason and given choices. When they answered ‘because I do not perform Pap smear’, we coded 1. For all other answers (did not think of it, smear taken recently by other professional, patient refused, patient would have been embarrassed, other, I don’t know) we coded 0 (Fig. 1).

### Independent variables

Covariates were GP personal characteristics such as sex, age, opinion towards vaccination generally and Human Papilloma Virus (HPV) vaccination specifically, whether they suggest vaccination against HPV and whether they are pleased with cooperation in psychology; and organizational characteristics such as regulated/unregulated fees, solo or group practice, using electronic medical records, practice of complementary and alternative medicine (CAM), number of daily consultations, participation in professional networks, having a role of trainer or internship supervisor, and other activities outside the physician’s office.

Covariates regarding county of practice (hereafter called county characteristics) were extracted from the National Institute of Statistics and Economic Studies (*Insee*) and the Institute for Research and Information in Health Economics (*Irdes*) databases and added to GP Health Barometer according to GP’s geographical location. We used medical demographic characteristics such as GPs and gynaecologist density, average time to access a GP and a gynaecologist, existence of an organized CCS program. We used socio-economic characteristics such as poverty rate (poverty line set at 60% of median standard of living) and poverty gap index (calculated as follows: (poverty line - median standard of living of the poor population) / poverty line; this indicator estimates the depth of poverty by considering how far, on average, the poor are from that poverty line).

The choices of county characteristic coding (in categories, linear or quadratic) was determined using graphical representation of the rate of GPs never practicing Pap smear as a function of each covariate.

### Statistical analysis

Data were clustered with several GPs (first level) for the same county (second level). Mixed logistic models with a random intercept [24] were used to take this hierarchical structure into account. Firstly, GP personal and county characteristics were tested in univariate analysis and selected for multivariate analysis if they had *p* ≤ 0.2. Secondly, selected GP characteristics were introduced in a multivariate model adjusted on GPs’ age and sex. We performed a manual backward stepwise procedure to retain only characteristics with *p* ≤ 0.05. The resulting model was named GP model (i.e. model containing only GP characteristics). Thirdly, county characteristics selected in univariate analysis were introduced in the GP model. We performed backward stepwise procedure for county characteristics only to retain characteristics with *p* ≤ 0.05 to obtain the final model.

Finally, the percentage of reduction (PR) of the inter-county variance between GP model (σ_i_^2^) and the final model (σ_f_^2^) was calculated using the following formula: PR = (σ_i_^2^ − σ_f_^2^)/σ_i_^2^. This calculation allows us to quantify the effect of county characteristics on the variations in the rate of GPs never performing Pap smear between counties, independently of an effect due to the composition of the GPs. For some counties, rates might have been better because they had GPs with characteristics associated with better involvement in Pap smear (more women GPs for instance) and not because of county characteristics.

All analyses were performed using SAS 9.4.

## Results

In our analyses, 70.7% of physicians were men (Table 2). Mean age of GPs was 50.6 years (standard deviation = 8.7). Over one third of doctors (34.7%) declared not performing CCS. The proportion of GPs who don’t perform CCS varied significantly between counties (inter-counties variance = 0.71, *p* ≤ 0.0001), ranging from 12 to 57% for the 2.5th and 97.5th percentile of the distribution.

**Table 2:** GP characteristics associated with never performing Pap smear - Univariate analysis (*N* = 1063)

GP characteristics	n (%)	% of GPs never performing Pap smear	OR	[95%CI]	p
Sex					
Female	312 (71)	22.8	1		< 0.0001
Male	751 (29)	39.7	2.71	[1.93–3.80]	
Age (years)					
≤ 40	161 (15)	34.2	1.13	[0.75–1.69]	0.77
[40–50]	330 (31)	35.8	1.10	[0.80–1.50]	
> 50	572 (54)	34.3	1		
Fee regulation					
Regulated	952 (90)	32.6	1		0.001
Unregulated	110 (10)	53.6	2.11	[1.35–3.31]	
Other activities outside physician’s office					
Yes	344 (32)	29.1	1		0.01
No	718 (68)	37.5	1.48	[1.09–2.01]	
Practice type					
Group	554 (52)	31.2	1		0.02
Solo	508 (48)	38.6	1.40	[1.06–1.85]	
Electronic medical records					
Yes	822 (77)	31.9	1		0.001
No	240 (23)	44.6	1.71	[1.23–2.36]	
Acupuncture					
Regularly	51 (5)	58.8	3.62	[1.90–6.92]	< 0.0001
Occasionally	28 (3)	32.1	0.73	[0.31–1.75]	
Never	983 (92)	33.6	1		
Homoeopathy					
Regularly	137 (13)	46.0	2.03	[1.31–3.13]	0.01
Occasionally	463 (44)	33.5	1.11	[0.82–1.50]	
Never	460 (43)	32.8	1		
Other CAM^a^					
Regularly	100 (9)	48.0	2.02	[1.27–3.22]	0.01
Occasionally	118 (11)	39.0	1.20	[0.77–1.85]	
Never	844 (80)	32.6	1		
Take part in health network					
Yes	420 (40)	29.1	1		0.001
No	639 (60)	38.5	1.67	[1.24–2.24]	
Pleased with the cooperation in psychology					
Yes	319 (30)	42.0	1		0.01
No	738 (70)	31.7	0.68	[0.50–0.92]	
Trainer or internship supervisor					
Yes	197 (19)	27.9	1		0.03
No	866 (81)	36.3	1.52	[1.05–2.21]	
Good opinion towards vaccination					
- generally					
Much	837 (79)	31.5	1		< 0.0001
Rather	199 (19)	44.2	1.68	[1.19–2.37]	
Rather not	22 (2)	63.6	4.05	[1.54–10.66]	
- against HPV^b^					
Much	616 (58)	33.0	1		0.09
Rather	322 (31)	36.3	1.22	[0.89–1.67]	
Rather not	81 (8)	34.6	1.11	[0.65–1.89]	
Not at all	35 (3)	51.4	2.5	[1.18–5.30]	
Suggests vaccination against HPV^b^					
Always	542 (52)	29.0	1		0.001
Often	341 (33)	38.4	1.61	[1.18–2.20]	
Sometimes	107 (10)	41.1	1.67	[1.04–2.68]	
Never	58 (5)	50.0	2.61	[1.43–4.76]	

^a^CAM: Complementary and alternative medicine

^b^HPV: Human Papilloma Virus

**Fig. 2:**
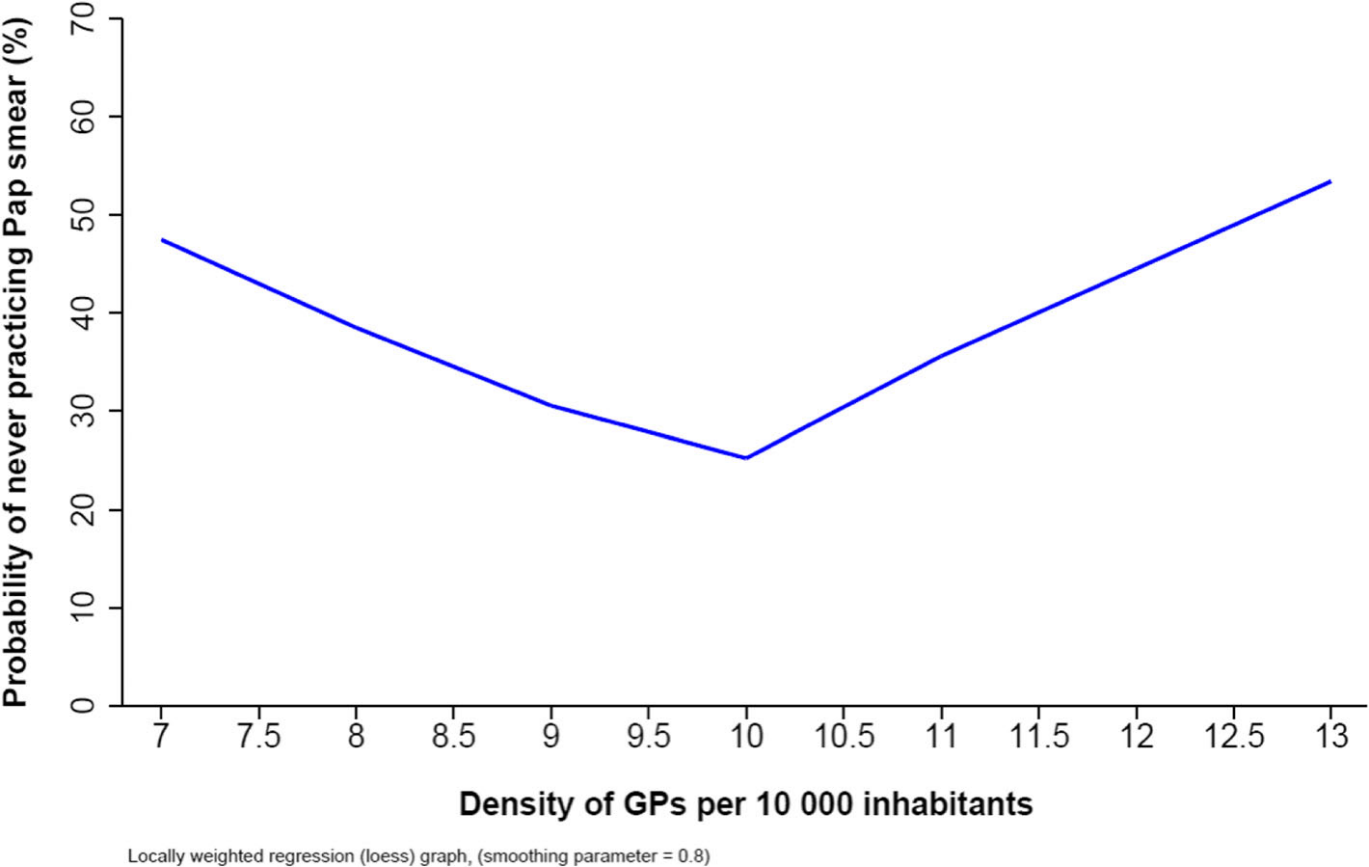
Association between GPs’ density for 10,000 inhabitants and not performance of CCS

In univariate analysis, almost all GPs’ characteristics were associated with not performing CCS. Men GPs were more likely not to perform CCS. GPs were also more likely not to perform CCS when they did not use electronic medical records, didn’t participate in healthcare networks and had a regular practice of CAM (Table 2). Almost all county characteristics (described in Table 3) were significantly associated with not performing CCS in univariate analysis (Table 4). GPs working in a county with high poverty indicators were more likely not to perform CCS: GPs declared never performing Pap smear all the more frequently that they worked in a county with higher poverty gap index. The association with a poverty rate below the national average was at the border of the statistical association. Health care availability and access characteristics were also associated with no performance of CCS: GPs declared never performing Pap smear all the more frequently that they worked in a county with higher gynaecologist density. GPs working in a county where time to access a gynaecologist was less than 15 mins (i.e. below the national average time) and in a county where time to access a GP was less than 1 mins (i.e. below the national average time) were more likely not to perform CCS. Graphic representation of the association between not performing CCS and GP density in the county was piecewise linear with a V shape (Fig. 2). GPs working in counties where GPs density was furthest away from the breakpoint at 9.5 GPs per 10,000 inhabitants (i.e. approximatively the national average) were more likely not to perform CCS (i.e. in counties with very low or very high GP density).

**Table 3:** French county (*département*) characteristics

	Min	Median (IQR^*^)	Max
Poverty rate (%)	8.2	14.3 (12.3–15.6)	24.8
Poverty gap index (%)	15.7	18.8 (18–19.5)	24.1
GPs density (GPs per 1000 inhabitants)	0.7	1 (0.8–1.1)	1.3
Time to go to GPs (minutes)	0	1.4 (0.8–1.9)	3.8
Gynecologists density (Gyn. per 100,000 women)	4	16 (13–19)	55
Time to go to gynecologists (minutes)	0	14.8 (11.3–18.2)	33.3

*IQR: Interquartile range

**Table 4:** County characteristics associated with never performing Pap smear - Univariate analysis (N = 1063)

County characteristics	% of GPs never performing Pap smear	OR	[95%CI]	p
*Categorical*				
Poverty rate above the national average^*^				
Yes	40.7	1.59	[0.99–2.53]	0.054
No	29.1	1		
Time to go to GPs				
> 1 min^#^	26.8	1		0.01
≤ 1 min	39.6	1.91	[1.20–3.05]	
Time to go to gynecologists				
> 15 min^#^	18.9	1		< 0.0001
≤ 15 min	39.2	2.97	[1.78–4.93]	
Organized CCS program				
Yes	25.4	0.73	[0.24–2.25]	0.58
No	35.4	1		
*Linear (1 unit of increase)*				
Poverty gap index (1 percentage point)	–	1.39	[1.22–1.58]	< 0.0001
Gynecologists density (1 Gyn. per 100,000 women)	–	1.07	[1.04–1.1]	< 0.0001
*Piecewise regression (1 unit of increase)*				
GPs density (1 GPs per 10,000 inhabitants)				
Slope below 9.5 GPs per 10,000 inhabitants^#^	–	0.53	[0.37–0.77]	0.001
Slope above 9.5 GPs per 10,000 inhabitants	–	1.55	[1.24–1.94]	0.0001

GP: General Practitioner

*national average = 14%

^#^approximately the national average

In the final model (Table 5), younger GPs and men GPs were more likely to report not performing Pap smear. Not performing CCS also remained associated with not using electronic medical records, practising homeopathy and acupuncture and not participating in a healthcare network. Concerning county characteristics, poverty rate above the national average was associated with not performing CCS. A higher density of gynaecologists and access time to a gynaecologist below 15 mins was associated with not performing the screening. GP density up to the national average was negatively associated with no performance of CCS: for GPs working in counties with GP density below 9.5 per 10,000 inhabitants, increase in GP density was associated with a lower likelihood not to perform CCS.

**Table 5:** GP and county characteristics associated with never performing CCS - Final model (*N* = 1013)

	OR	[95% CI]	p
*GP characteristics*			
Male sex	3.92	[2.67–5.76]	< 0.0001
Age *(Ref. > 50 years)*			
[40–50]	1.68	[1.18–2.38]	0.0008
≤ 40	2.21	[1.39–3.53]	
No electronic medical records	1.78	[1.23–2.56]	0.002
Acupuncture *(Ref. Never)*			
Regularly	2.95	[1.34–6.50]	0.007
Occasionally	0.49	[0.18–1.34]	
Homeopathy *(Ref. Never)*			
Regularly	2.10	[1.26–3.52]	0.02
Occasionally	1.25	[0.90–1.73]	
Not taking part in health network	1.72	[1.25–2.36]	0.0008
*County characteristics*			
Poverty rate above the national average^a^	1.66	[1.09–2.54]	0.02
GPs density *(unit = 1 per 10,000 inhabitants)*			
Slope below 9.5 GPs per 10,000 inh. ^b^	0.52	[0.37–0.74]	0.0003
Slope above 9.5 GPs per 10,000 inh.	1.17	[0.92–1.48]	0.2
Gynecologists density *(unit = 1 Gyn. per 100,000 women)*	1.06	[1.03–1.10]	0.0003
Less than 15 min time to go to gynecologists	2.02	[1.20–3.41]	0.008

Characteristics integrated and then removed: fee regulation, other activities outside physician’s office, practice type, other CAM, pleased with the cooperation in psychology, pleased with the cooperation in psychology, good opinion towards vaccination generally, good opinion towards vaccination against HPV, suggests vaccination against HPV, trainer or internship supervisor, intensity of poverty, time to go to GPs

^a^national average = 14%

^b^approximately the national average

The reduction in the inter-county variance because of county characteristics (poverty, availability and time access to healthcare provides) was 93%. In other words, if all counties had the same poverty rate, medical density and access time to gynaecologists, the variability between counties in the rate of GPs never performing Pap smear would almost disappear.

## Discussion

### Summary

More than one third of the GPs of our sample declared never performing Pap smear. GPs working in counties with fewer GPs per inhabitants than the national average, in counties with an easier access to a gynaecologist (i.e. higher density and first gynaecologist reachable in less than 15 min) or with a poverty rate above the national average were more likely not to perform CCS. These contextual characteristics explain most of the differences between counties in terms of GP never performing Pap smear rate. In addition to already-known GP characteristics associated with no performance of CCS, practicing CAM (acupuncture or homeopathy) was also identified.

### Comparison with literature on rates of CCS

These context-specific characteristics associated with no performance of CCS by GPs are similar to characteristics associated with low CCS rates in the literature. Many studies found lower screening rates in areas of residence with lower healthcare availability [25–30] and longer distance or travel time to physicians [31]. Women living in socially advantaged areas were also more up-to-date [8, 25–28, 32–39].

### Interpretations

We found that GPs’ practice was influenced by both gynaecologist density and access time to a gynaecologist. There is thus a two-fold component in access: on the one hand practitioners’ density refers to availability of doctors with regard to the population to be screened, and on the other hand geographical accessibility corresponds to their more or less homogeneous distribution within the county.

GPs working in a county with low GPs’ density were more likely not to perform CCS than their colleagues working in a county where GPs’ density was near the national average. We could distinguish two different dynamics affecting CCS performance, based on county-level GP density: in areas with a very low GP density, an increase in GP density meant an increased likelihood to perform Pap smear. In these GP-poor areas, GPs may have limited time for preventive care such as CCS, but an increase in GP density might make it possible. These areas with few GPs might be less attractive for practitioners in general, including gynaecologists, there is therefore a need for GPs to undertake CCS. However, in areas with higher GP density, an increase in GP density meant a decreased likelihood to perform Pap smear. These might be more attractive and wealthy areas, benefitting from a higher gynaecologist density: therefore, it may appear less crucial to GPs to perform CCS, as other practitioners can undertake them.

A poverty rate below the national average was associated with no performance of CCS by GPs. Since the least wealthy counties are also those with the lowest physician density, there is a cumulative effect of poverty in terms of financial and health resources on screening rates.

Finally, GPs practicing CAM (acupuncture or homeopathy) were more likely not to perform CCS. Although we couldn’t find any comparable results in the literature, we suggest that GPs with a CAM practice may have different patients and specific activities, focused more on CAM and less on primary care.

All these county characteristics are decisive to understand the differences between counties. Indeed, they explain almost all of the inter-county variability in the rate of GP never performing Pap smear.

### Study limitations and strengths

Our study has some limits. First, to analyse the role of economic and demographic contextual elements - such as levels of poverty and physician density - on the performance of Pap smear, we only had access to data at the county level: we knew in which county each GP worked and we matched this information with economic and demographic information available for each county from the French National Institute for Statistical and Economic Studies (INSEE). Our main difficulty was that French counties are rather large entities (median surface area = 5880 km^2^) that can be heterogeneous in terms of economic indicators and physician density. Our analyses would have gained in precision with more precise information on GPs’ location, for example at the municipality level.

Second, our data are quite old. Barometers are cross-sectional surveys repeated every 3 yrs, unfortunately GPs’ geographical location was available only for the 2009 Barometer dataset.

Third, even if the proportion of GPs who do not perform CCS is consistent with other studies conducted in France [19, 40], it may be underestimated. For example, some GPs could be embarrassed to declare never performing the screening and may have given another reason leading to measurement bias regarding our variable of interest. However, because the survey inquired about a large number of issues, GPs’ answers may be less subject to social desirability bias than surveys focusing only on CCS. Additionally, we considered that GPs who declared not performing CCS for their last seen patient aged 50 to 60 years would not have responded differently for a younger patient.

Fourth, the lack of significance of organized CCS programs may be due to a lack of statistical power. In 2009, there were only three counties with such a program in continental France, which may explain the absence of association.

Finally, various individual elements such as a lack of training, difficulties in the management of intimacy or the potential preference of women to discuss gynecological problems with specialists, have already been identified as limiting the practice of Pap smears in general practice [14, 41, 42]. They could not be analyzed in this work but could influence the disinvestment of general practitioners in CCS.

The current study provides new information about the influence of medical demography and socio-economic environment on GP’s not performing CCS. It is one of the few studies focused on CCS providers and not on woman receiving CCS [13, 20, 21]. Moreover, the use of multilevel analysis allowed us to correctly estimate associations between GPs’ practices, GPs’ individual characteristics and context-specific elements of GPs’ practice. The generalization of our results is more or less easy depending on the country and the coexistence of several types of screening providers. But regardless of the national context, it seems that the preventive practices of general practitioners, or at least some of them, are influenced by the environment in which practitioners work.

### Implication for practice

GPs who work in high poverty-rate areas and in poor GP-density areas were more likely not to perform CCS. GPs’ working in these areas should be informed of this situation and of the possibility of limiting their investment in screening by referring their patients to the medical laboratory for Pap smears. Our results constitute a new illustration of the inverse care law: the supply of care is inversely proportional to health needs [43, 44]. In the case of Pap smear, medical resources seem to be inappropriately distributed in relation to needs. But in addition, low resources are also associated with lower investment by GPs in CCS, in a reinforcing effect.

Organized CCS was generalized in France in 2019. Women who have not been screened in the last 3 years will receive an invitation-letter for free screening. GPs will have an important place in this screening, to participate in the recruitment of women and convince them to participate, to carry out smears or refer them to other professionals who can ensure this sample or propose a self-sampling in search of HPV. Our results could make it possible to target GPs practicing in contexts that are unfavorable to smear testing in order to raise their awareness, offer them training or provide them with lists of samplers close to their place of exercise. Finally, it should be kept in mind that in order to significantly increase the screening rate, GPs will have to carry out Pap smears but also increase their screening activity to take a larger part in the coverage [40]. The two probably go hand in hand to the extent that an unusual or occasional activity struggles to be maintained on a long-term basis.

## Conclusions

Beyond the individual characteristics of GPs, physicians’ screening practices are shaped by their context of exercise: favorably when GPs become more involved in Pap smear in the absence of gynecologists and unfavorably when they disinvest it when they practice in poor areas or with a low GPs density. This type of analysis could be conducted regarding other types of care to further clarify the effect of practice settings on the care provided.

## Data Availability

GP Health Barometer survey are available from the *Santé Publique France* (https://www.santepubliquefrance.fr/) upon reasonable request.

## Abbreviations

CCS: Cervical cancer screening
GP: General practitioner
